# Metabolite-Centric Reporter Pathway and Tripartite Network Analysis of *Arabidopsi*s Under Cold Stress

**DOI:** 10.3389/fbioe.2018.00121

**Published:** 2018-09-11

**Authors:** Ibrahim Koç, Isa Yuksel, Gustavo Caetano-Anollés

**Affiliations:** ^1^Department of Molecular Biology and Genetics, Gebze Technical University, Gebze, Turkey; ^2^Department of Bioengineering, Gebze Technical University, Gebze, Turkey; ^3^Department of Crop Sciences, University of Illinois, Urbana, IL, United States

**Keywords:** reporter metabolite, reporter pathway, cold stress, pathway analysis, microarray, network modularity, power law

## Abstract

The study of plant resistance to cold stress and the metabolic processes underlying its molecular mechanisms benefit crop improvement programs. Here we investigate the effects of cold stress on the metabolic pathways of *Arabidopsis* when directly inferred at system level from transcriptome data. A metabolite-centric reporter pathway analysis approach enabled the computation of metabolites associated with transcripts at four time points of cold treatment. Tripartite networks of gene-metabolite-pathway connectivity outlined the response of metabolites and pathways to cold stress. Our metabolome-independent analysis revealed stress-associated metabolites in pathway routes of the cold stress response, including amino acid, carbohydrate, lipid, hormone, energy, photosynthesis, and signaling pathways. Cold stress first triggered the mobilization of energy from glycolysis and ethanol degradation to enhance TCA cycle activity via acetyl-CoA. Interestingly, tripartite networks lacked power law behavior and scale free connectivity, favoring modularity. Network rewiring explicitly involved energetics, signal, carbon and redox metabolisms and membrane remodeling.

## Introduction

Environmental stresses constrain the growth and production of plants. Abiotic stresses include drought, cold, salt, and osmotic shock and biotic stresses include wounding and pathogen attack. Both kinds of stresses can form reactive oxygen species (ROS) leading to oxidative damage of proteins, nucleic acids, and lipids (Mittler, 2002). Stress tolerance and physiological adaptations mitigate the effects of stress. These processes are mediated by differential gene expression regulating the activity of a number of metabolic pathways and their associated metabolites (Lakshmanan et al., 2015).

Metabolic networks consist of collections of gene-encoded enzyme-catalyzed reactions that convert metabolites that transfer energy and matter along reaction pathways. The exploration of metabolic changes in plants that occur at system level in response to environmental perturbations can be modeled with systems biology approaches. Currently, modeling all components and interactions of a biological system is not realistic due to the large number of parameters that need to be considered (Cakir et al., 2006). One shortcut is to model metabolic activities from transcriptome data to reveal plant responses to abiotic stress (Liu et al., 2016). Many genome-scale models have been developed for model plants and plants of agronomic importance, including *Arabidopsis* (de Oliveira Dal'Molin et al., 2010) and *Chlamydomonas reinhardtii* (Dal'Molin et al., 2011), rice (Lakshmanan et al., 2015), and maize (Saha et al., 2011), facilitating global and realistic documentation of perturbation effects in plants.

Omics scale data enables global metabolic overviews. However, understanding cellular physiology requires gaining information of the metabolome and the complexity and high connectivity of metabolic reactions (Nielsen, 2003; Cakir et al., 2006). Metabolic network, sub-network and reporter metabolites have been identified using well-annotated biological data (Patil and Nielsen, 2005). The biological knowledge obtained from these genomic scale approaches is translated into many potential applications, such as engineering agronomic traits and designing stress response gene circuits in crops. Understanding plant response mechanisms requires comprehensive integration of all the outputs of multi-omics approaches (Tatli et al., 2015). In recent years, there have been many profiling studies of the transcriptome, proteome, and metabolome focused on how plants interact with the environment when subjected to stress (Rensink et al., 2005; Budak et al., 2013; Kim et al., 2015; Koç et al., 2015c). However, uncovering genome scale metabolic networks comprising biochemical reactions from qualitative experimental proteomic and metabolomics data is difficult due to the limited sensitivity of current technologies (Yue et al., 2014).

Cold stress affects agricultural productivity in cold regions of the world. Numerous physiological and molecular changes occur during cold acclimation. Physiological changes include chilling injury and growth retardation, and molecular changes include gene and miRNA regulation (Koç et al., 2015a). Here we study the cold stress condition at different time points. We use transcriptome data to gain insight into changes of key metabolites and pathways of *Arabidopsis*. The Gene Set Enrichment (GSE) method is one of several approaches capable of revealing significant operating pathways from microarray and RNAseq data. These methods identify statistically significant gene sets that are differentially expressed in the gene or pathway lists. While they are often sufficient, there are some biological cases where only few differentially expressed genes are found amid large perturbations. Examples include diagnosis-relapse and tumor tissues (Staal et al., 2003; Yang et al., 2008; Tegge et al., 2012; Akkiprik et al., 2015). Although GSE provides pathway-level views, the general approach still evaluates each gene individually (Tegge et al., 2012). The approach does not describe how genes coordinate their activities within the pathways. Here we take advantage of an algorithm that maps metabolic gene expression by identifying the most significantly changed reporter metabolites using neighboring genes and gene-metabolite association. This reporter metabolite analysis, which has been successfully used in organisms such as yeast, *Arabidopsis* and rice (Çakir, 2015; Kim et al., 2015; Lakshmanan et al., 2015), elucidates the metabolic pathway regulation of cold stress in *Arabidopsis*.

## Materials and methods

### Data

The gene expression data of *Arabidopsis* under different abiotic stresses was retrieved from the GEO database of NCBI (ncbi.nlm.nih.gov/geo/). The data comprises cold stress ID series GSE5620 (Control plants) and GSE5621 (Cold stress). These datasets were from experiments carried out using Affymetrix ATH1 microarray chip technology, which included an array of over 22,000 genes (Kilian et al., 2007). In these samples, seeds of *A. thaliana* Wild Type (col-0) were sown on Magenta boxes containing MS-Agar media and grown as previously described (Kilian et al., 2007). Stress treatment was applied at day 16. In the present study, we investigated the stress response by selecting samples from a transcriptome data set that was sufficiently complete and time-resolved (3, 6, 12, and 24 h of cold treatment). The data correlation of the biological replicates for the cold stress experiment was over 0.95 (Kilian et al., 2007). Initially, data were quantile normalized across all samples, log_2_ transformed based on each time point of control, and processed with Principal Component Analysis (PCA) to reduce data dimensionality and help identify outliers. We extracted metabolic genes from AraCyc and then structured a custom-based Excel query to identify metabolic genes of interest within the Affymetrix ATH1 gene information file. The resultant metabolic gene set included 4,730 genes.

### Metabolite-centric reporter pathway analysis (RPA^m^)

First, we constructed a draft consensus of an *Arabidopsis* genome scale network by collecting annotated metabolic genes and their biochemical reactions from AraCyc v.14 (Mueller et al., 2003). Although the AraCyc model, which contains 3,225 reactions, 5,276 enzymes, 2,802 metabolites, and 542 pathways, the draft network was manually re-checked for the presence of reactions in *Arabidopsis* using KEGG (Kanehisa and Goto, 2000), wikipathways (https://www.wikipathways.org), and especially UniProt (https://www.uniprot.org). In particular, we focused on significantly changed metabolites and pathways for accuracy. Differentially expressed genes between control and stress samples were identified in the Matlab environment using the *ttest2* command based on two-sample *t*-test. Subsequently, *P-*values were calculated for each gene.

We constructed three files, a *P*-value data file, a gene metabolite association file, and a pathway metabolite association file for MATLAB. In RPA^m^, a metabolite-score must be computed first by the Reporter Metabolic Centric algorithm (Oliveira et al., 2008). The approach depends on the underlying structure of the data and the basic assumptions of the gene expression analysis technology. Since a gene is usually represented by several sets of probes in for example microarray analysis, the significance of differentially expressed genes was assessed by assigning a *P*-value to each probe set and taking into account the variation within the groups being compared; the *P*-value for a differentially expressed gene returns the value from the top probe set following established probe set ranks. For reporter metabolite analysis, the genes were linked to the corresponding metabolites via controlled reactions with an algorithm that takes as input *P*-values of gene expression data and the topology of bio-molecular interaction networks represented as a graph (Ideker et al., 2002). *P*-values were then assigned to relevant enzymes and converted to Z scores with an inverse cumulative distribution function (θ^−1^). The use of this distribution function converts uniformly distributed *P*-values for each probe set into normally distributed standard *Z* score random variables. For isozymes, the lowest *P*-values of isozymes were selected.

Once each enzyme was scored with a *Z*-value, a *Z*_*metabolite*_ score was calculated for each metabolite as an aggregate *Z* score of its *k* neighboring genes in the metabolic network scale. In this step, the *Z* scores of genes connected to a metabolite in the metabolic model were averaged to obtain a final *Z* score for the metabolite (Cakir et al., 2006).

$$\begin{array}{l} Z_{metabolite}=\;\frac{1}{\sqrt{k}}\overset{k}{\underset{i=1}{\sum }}Z_{i} \end{array}$$

Subnetworks of all sizes are comparable under this scoring system. If the *Z*_*gene*_ scores are independently drawn from a standard normal distribution, the aggregate *Z*_*metabolite*_ score will also be distributed according to a standard normal distribution, independent of *k* (Ideker et al., 2002). A high *Z*_*metabolite*_ score indicates a biologically active subnetwork.

In order to evaluate the significance of metabolite scores, the score of each metabolite was corrected by calculating the mean (μ_*k*_) and standard deviation (σ_*k*_) of a background *Z* score distribution resulting from 10,000 rounds of random sampling of *k* metabolites of metabolic networks (Väremo et al., 2013). The number of random samples of the distribution was determined by checking the sensitivity of the resulting background scores relative to the number of random samples. The *Z* score can be considered a parameter of statistical significance (typically *Z* = 1.96 corresponds to *P* = 0.05). Furthermore, the reporter metabolite analysis is similar to the method of Stouffer. However, the statistical parameters of the gene set were corrected for the background distribution prior to the estimation of significance (for details see Oliveira et al., 2008; Väremo et al., 2013).

$$\begin{array}{l} {Z^{\prime }}_{metabolite}=\;\frac{Z_{metabolite}\;{-\;u}_{k}}{\sigma_{k}} \end{array}$$

**Figure 1:**
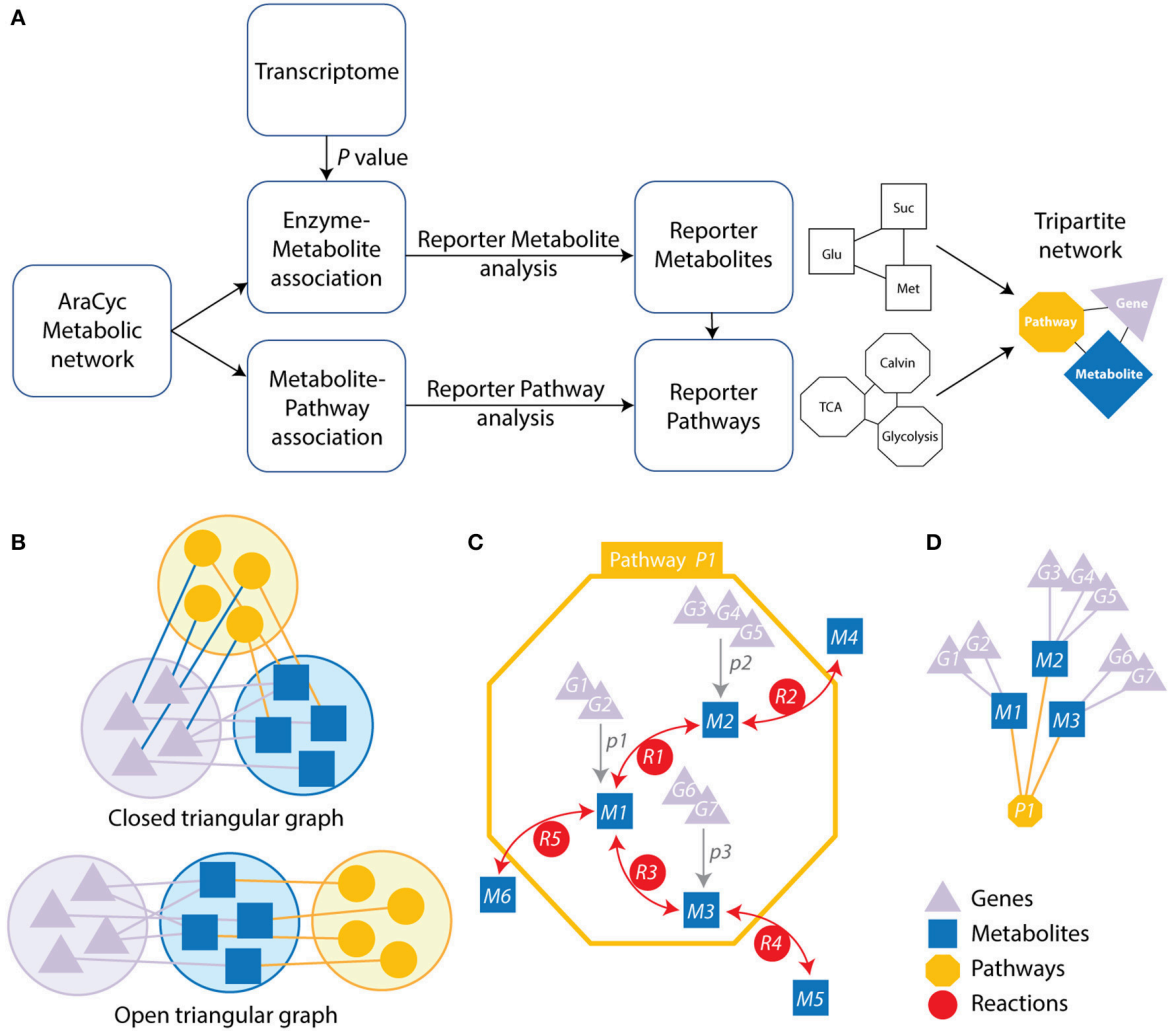
Strategy and schematic representation of the metabolic-centric reporter pathway analysis (RPA^m^) and its visualization with a tripartite network. **(A)** The flow diagram describes the computational strategy of the reporter pathway analysis and the integration of transcriptome data. *P*-values for differentially expressed genes are mapped onto metabolites associated genes. Each enzyme is assigned a score based on the average of the *P*-values of the probe sets representing the corresponding gene. Minimum *P*-values are chosen for a reaction catalyzed by an enzyme complex or a set of isoenzymes. *P*-values are then converted into Z scores and corrected with a background Z score distribution. The resulting scores are linked to related pathways, which are represented with a tripartite network. **(B)** Open and closed tripartite networks always connect nodes of different sets, with or without restrictions in how sets are connected with each other, respectively. **(C)** A pathway (yellow octagon) defines a set of reactions (red circles labeled with letter R) connecting metabolites (blue squares labeled with M). Genes (purple triangles labeled with G) can be associated to specific metabolites (gray arrows) when these are collectively significantly expressed at some *P-*value (labeled with *p*) over some threshold. **(D)** The resulting open tripartite network of genes, metabolites and pathways derived from the associations of **(C)**.

Here, the analysis of a “corrected” *Z*-value, ${Z^{\prime }}_{metabolite}$ is the reporter metabolite analysis (Patil and Nielsen, 2005). The final *Z* score was then transformed into a *P* value using the normal cumulative distribution and assigned to each metabolite (Figure 1) in order to detect those that are significant at *P* ≤ 0.05 level, which represent *reporter metabolites*.

Note that we removed some metabolites from the reporter metabolite analysis, including proton, water, oxygen molecule, NADH, NAD^+^, NADPH, NADP^+^, ATP, ADP, AMP, GTP, GDP, UDP, CoA, FAD, diphosphate, carbon dioxide, carbon monoxide, phosphate, ammonia, hydrogen peroxide, oxidized electron acceptor, and reduced electron acceptor. Their role in many reactions bias metabolite measurements (Kim et al., 2015).

Each metabolic pathway is similarly scored based on its metabolites. Scoring of significant pathways is based on *P-*values and *Z* scores of the corresponding metabolites following 10,000 iterations of the sampling algorithm.

$$\begin{array}{l} Z_{pathway}=\;\frac{1}{\sqrt{n}}\overset{n}{\underset{metabolite=1}{\sum }}Z_{metabolite} \end{array}$$

*n* is the number of metabolites associated with the pathway. A significance level of *P* ≤ 0.05 was used as a threshold to identify reporter pathways. Note that the *Z*_*pathway*_ score is corrected for the background distribution as calculated in ${Z^{\prime }}_{metabolite}$. Pathways with significant ${Z^{\prime }}_{metabolite}$ scores are called reporter pathways (Çakir, 2015).

We used the genome scale connection of genes and metabolites that are embedded in pathways to construct a tripartite network. They are represented in the graph of Figure 1, which illustrates these connections. This atypical network has vertices describing significantly changed genes, metabolites, and pathways and edges representing the connectivity of significantly changed vertices. The network was used to study time resolved responses of *Arabidopsis* to cold stress.

### Network analysis

The biological networks of genes, metabolites and pathways were visualized with Cytoscape 3.2 (Shannon et al., 2003). Clustering analysis was performed with the ClusterViz plugin using the MCODE algorithm, detecting clusters with default cutoffs. Scale free network behavior was examined using log-log plots, i.e., log of *P(k)* vs. log of *k*. The exponent γ of the power law [*P(k)* ~ *k*^−γ^] and the determination coefficient (*R*^2^) were derived from linear regression models. γ is the negative of the slope of the log linear model. *R*^2^ explains how much of the data fits the linear model. The scale free behavior of the network should be considered strongly supported when both γ and *R*^2^ are high. The Kolmogorov-Smirnov (KS) fit statistic compares the fitted distribution with the input degree vector and the KS *P*-value (Newman, 2005; Clauset et al., 2009). The null hypothesis corresponds to data drawn from the power law distribution. We determined the maximum log likelihood of the fitted parameters. We also determined if the power law fit pattern was continuous. We created reference networks with Barabási methods using the *R*'s igraph package (Csardi and Nepusz, 2006) to simulate a power law for the statistical analysis of networks. Modularity analyses were performed by using *Fast Greedy Community* (FGC) and *Newman-Girvan* (NG) algorithms (Clauset et al., 2004; Newman and Girvan, 2004). The NG algorithm iteratively computes the “betweenness” of edges, i.e., the number of shortest paths that run through an edge; the network is decomposed into communities by systematically removing edges and recalculating betweenness measures after each removal (Newman and Girvan, 2004). The FGC hierarchical agglomeration algorithm runs in time O(*md* log *n*) ~ O(*n* log^2^ *n*), with *m, n*, and *d* representing edges, vertices and the depth of the dendrogram describing the structure of the community, respectively (Clauset et al., 2004). In addition, time resolved tripartite networks were overlaid and visualized using CompNet software (Kuntal et al., 2016).

## Results and discussion

### Reporter pathway and tripartite network analysis

We explored the effect of cold stress on the metabolic networks of *Arabidopsis* with the RPA^m^ approach by: (i) analyzing differentially expressed genes in two transcriptome datasets (GSE5620 and GSE5621), (ii) associating these genes to metabolites, metabolic reactions, and metabolic pathways, and (iii) visualizing genes, metabolites and pathways using a tripartite graph representation (Figure 1A). The central data that is mined is the transcriptome. We assume that the differential expression of metabolic genes at a biological endpoint indicates their active involvement in core metabolic functions and/or their regulation.

Tripartite graphs are a special case of *k*-partite graphs with *k* = 3. *k*-partite graphs have nodes (graph vertices) that can be divided (partitioned or colored) into *k* disjoint sets (partitions or colors) and connections (graph edges) that always connect nodes belonging to different sets. Closed tripartite graphs do not impose restrictions on the tripartite structure of connected nodes (all sets can connect to each other) (Figure 1B). In contrast, open tripartite graphs do not allow a circular connecting structure. A gene-metabolite-pathway tripartite network describes the association of genes to metabolites at some minimum *p*-value and the association of metabolites to metabolic pathways through reactions (Figure 1C). We describe these associations with an “open” tripartite graph representation of genes (*G*_*i*_), metabolites (*M*_*i*_), and pathways (*P*_*i*_), in which allowed connections are only between *G*_*i*_ and *M*_*i*_ nodes and between *M*_*i*_ and *P*_*i*_ nodes (Figure 1D). One benefit of open tripartite graphs is that they can be decomposed into one-mode and two-mode (bipartite) graph projections to improve visualization. For example, our tripartite graph can be transformed into a more coarse-grained bipartite graph of *G*_*i*_ and *M*_*i*_ nodes by excluding pathway *P*_*i*_ node information, i.e., how metabolites connect many different operating pathways. Alternatively, exclusion of gene *G*_*i*_ information produces a bipartite graph of *M*_*i*_ and *P*_*i*_ nodes.

An initial exploration of the differential expression of over 22,000 genes with PCA analysis showed samples clustering into groups and a time resolved response to cold stress occurring at 4 time points, 3, 6, 12, and 24 h of cold treatment (Figure S1). We also conducted PCA analysis using only metabolic genes. The projections of metabolic genes were consistently similar to those of all genes. These findings supported the use of the metabolic gene subset to study metabolic mechanisms behind stress perturbations in *Arabidopsis*.

Note that a reporter metabolite approach of our metabolite-centric RPA^m^ better represents the effects of environmental perturbations at network-level since it considers all connecting reactions that consume or produce the metabolites of the pathway (Çakir, 2015). Notably, the RPA^m^ algorithm does not require a priori knowledge of changes that are significant at the level of each node (e.g., the transcript of a gene) (Oliveira et al., 2008). In contrast, a reaction-centric pathway analysis takes into account only significantly changed gene transcripts which are involved in associated reactions. Note that genes that are not differentially expressed at significant levels can operate in coordination with others (Mootha et al., 2003). Thus, it is important to consider both the magnitude of the significance of their differential expression and their connectivity in the network (Çakir, 2015). Using the reporter metabolite algorithm, we detected reporter metabolites in *Arabidopsis* and found that 64, 68, 71, and 102 reporter metabolites were significantly (*P* ≤ 0.05) regulated in the 3, 6, 12, and 24 h cold acclimated plants, respectively (Table 1, Supplementary File 1). In turn, 79, 79, 103, and 94 reporter pathways were significantly regulated in the corresponding cold treatments (Table 2, Supplementary File 1). We identified several metabolic pathways regulated under cold stress, including glutathione redox reactions, sucrose biosynthesis, and the biosynthesis and degradation of starch, mannitol, xylan, sucrose, triacylglycerol, cellulose, 3-phosphoinositide, L-ascorbate, and trehalose. In contrast, GSE analysis led to the identification of 45, 58, 89, and 47 pathways in the 3, 6, 12, and 24 h cold acclimated plants, respectively (data not shown). These pathways were mainly related to redox, sugar, lipid, amino acid metabolisms, which are similar to those of RPA^m^. Remarkably, GSE analysis did not capture the TCA cycle at any time point, which is a known effect of cold stress (Kaplan et al., 2004).

**Table 1:** Top 20 reporter metabolites identified for each time point of cold stress are labeled with matching superscript letters (a, b, c, and d for 3h, 6h, 12h and 24h, respectively) and listed with associated *P-*values.

**Metabolites**	**3h^a^**	**6h^b^**	**12h^c^**	**24h^d^**
Alpha-D-mannose 6-phosphate^a^	0.00163	0.01447	0.63452	0.26521
Glutathione disulfide^a, c^	0.00305	0.07740	0.00786	0.07642
Trans-zeatin riboside diphosphate^a^	0.00345	0.13175	0.70004	0.72588
Trans-zeatin riboside triphosphate^a^	0.00345	0.13175	0.70004	0.72588
Glutathione^a, c^	0.00437	0.15692	000179	004358
Trans-zeatin riboside monophosphate^a^	0.00534	0.05419	0.31520	0.73948
1-phosphatidyl-1D-myo-inositol 4,5-bisphosphate^a, d^	0.00535	0.08122	0.02957	0.01341
Linoleate^a, b^	0.00859	0.00795	0.17382	0.29607
D-myo-inositol (1,4,5)-trisphosphate^a^	0.01007	0.15141	0.08069	0.24371
Alcohol^a^	0.01124	0.17401	0.05947	0.62327
3-aminopropanal^a^	0.01355	0.04838	0.35844	0.94092
Maltodextrin^a^	0.01437	0.04653	0.90617	0.11826
2-hydroxy-3-butenylglucosinolate^a^	0.01466	0.08343	0.32611	0.04110
Linoleoyl-CoA^a, b^	0.01480	0.02288	0.19277	0.76198
(+)-7-iso-jasmonoyl-L-isoleucine^a^	0.01521	0.21418	0.23448	0.55750
(3R, 7S)-12-hydroxy-jasmonoyl-L-isoleucine^a^	0.01521	0.21418	0.23448	0.55750
Alpha-D-glucose 1-phosphate^a^	0.01554	0.04642	0.02136	0.02105
Carboxylic ester^a^	0.01706	0.03445	0.08737	0.25514
L-arogenate^d^	0.01732	0.18897	0.33126	0.01905
Pectin^a^	0.01985	0.06709	0.06351	0.04816
Maltose^d^	0.07460	0.00197	0.01907	0.22904
Donor xyloglucan^b^	0.14066	0.00284	0.08807	0.01597
Donor xyloglucan with cleaved xyloglucanyl segment^b^	0.14066	0.00284	0.08807	0.01597
Acceptor xyloglucan^b^	0.14066	0.00284	0.08807	0.01597
Acceptor xyloglucan with xyloglucanyl segment^b^	0.14066	0.00284	0.08807	0.01597
Beta-lactam^b^	0.51370	0.00379	0.08762	0.97359
Substituted beta-amino acid^b^	0.51370	0.00379	0.08762	0.97359
Primary alcohol^b^	0.06146	0.00811	0.38011	0.06872
(1.4-alpha-D-galacturonosyl)(n+m)^b^	0.08196	0.00983	0.03184	0.07553
UDP-alpha-D-galacturonate^b^	0.13013	0.01012	0.04762	0.07452
Succinate semialdehyde^b^	0.60192	0.01215	0.42555	0.75903
Ethanol^b^	0.22968	0.01331	0.23421	0.13120
Long-linear glucan^b^	0.02613	0.01884	0.12721	0.53747
Formate^b, c^	0.36096	0.01919	0.01419	0.55658
(1,4-alpha-D-galacturonosyl)(n)^b^	0.05706	0.01932	0.04606	0.03385
Dimethylglycine^b^	0.07132	0.02212	0.12359	0.31639
Maltotetraose^b^	0.04192	0.02318	0.12423	0.21071
UDP-alpha-D-xylose^c^	0.12865	0.03932	0.00212	0.01164
Glycerophosphodiester^c^	0.16110	0.10380	0.00586	0.24715
(1,4-alpha-D-galacturonide)(n-1)^c^	0.17113	0.09248	0.00745	0.10762
(1,4-alpha-D-galacturonide)n^c^	0.17113	0.09248	0.00745	0.10762
XXXG xylogulcan^c^	0.23472	0.20916	0.00804	0.09012
1,4-beta-D-glucan^c^	0.15849	0.19719	0.00918	0.13785
GXGG xylogulcan^c^	0.15849	0.19719	0.00918	0.13785
Very-long-chain oxoacyl-CoA^c^	0.31195	0.27893	0.01025	0.06199
4-hydroxy-3-indolylmethyl-glucosinolate^c^	0.52259	0.21735	0.01204	0.08633
Quercetin 3-sulfate^c^	0.66214	0.29294	0.01237	0.45572
1-naphthol 6-O-malonylglucoside^c^	0.45960	0.30617	0.01371	0.30724
1-naphthol glucoside^c^	0.45960	0.30617	0.01371	0.30724
2-naphthol 6-O-malonylglucoside^c^	0.45960	0.30617	0.01371	0.30724
2-naphthol glucoside^c^	0.45960	0.30617	0.01371	0.30724
4-methylumbelliferone 6-O-malonylglucoside^c^	0.45960	0.30617	0.01371	0.30724
Very-long-chain 2,3,4-saturated fatty acyl CoA^c^	0.69164	0.19241	0.01396	0.15649
1,2-diglyceride^c^	0.25227	0.55788	0.01468	0.07611
L-allo-threonine^d^	0.78740	0.83498	0.37190	0.00119
Pectate^d^	0.03367	0.11425	0.02110	0.00783
Indole-3-butyrate^d^	0.35778	0.46690	0.30821	0.01113
indole-3-butyryl-glucose^d^	0.35778	0.46690	0.30821	0.01113
a 1,2-diacyl-sn-glycerol 3-phosphate^d^	0.03164	0.02348	0.06917	0.01260
8-methylthiooctyl glucosinolate^d^	0.65439	0.25748	0.10328	0.01351
3-methylthiopropyl-glucosinolate^d^	0.65439	0.25748	0.10451	0.01351
4-methylthiobutyl glucosinolate^d^	0.65439	0.25748	0.10451	0.01351
5-methylthiopentylglucosinolate^d^	0.65439	0.25748	0.10451	0.01351
6-methylthiohexylglucosinolate^d^	0.65439	0.25748	0.10451	0.01351
7-methylthioheptyl glucosinolate^d^	0.65439	0.25748	0.10451	0.01351
2-succinylbenzoate^d^	0.91763	0.62208	0.28534	0.01411
[(1->4)-beta-D-xylan](n)^d^	0.38632	0.15860	0.09204	0.01426
[(1->4)-beta-D-xylan](n+1)^d^	0.38632	0.15860	0.09204	0.01426
glycogen^d^	0.37403	0.59350	0.78145	0.01481
3-methylthiopropyl-desulfoglucosinolate^d^	0.99274	0.76625	0.14823	0.01593
4-methylthiobutyldesulfoglucosinolate^d^	0.99274	0.76625	0.14823	0.01593
5-methylthiopentyldesulfoglucosinolate^d^	0.99274	0.76625	0.14823	0.01593

**Table 2:** Top 20 reporter pathways identified for each time point of cold stress are labeled with matching superscript letters (a, b, c and d for 3h, 6h, 12h and 24h, respectively) and listed with associated *P-*values.

**Pathway names**	**3h^a^**	**6h^b^**	**12h^c^**	**24h^d^**
3-phosphoinositide biosynthesis^d^	0.00480	0.01341	0.05976	0.00001
6-hydroxymethyl-dihydropterin diphosphate biosynthesis I^a^	0.00185	0.06439	0.04810	0.45753
Ascorbate glutathione cycle^a, d^	0.00019	0.11972	0.00085	0.00087
Calvin-Benson-Bassham cycle^c, d^	0.11022	0.15765	0.00034	0.00280
Cellulose biosynthesis^d, d^	0.01291	0.01685	0.01031	0.00106
Choline biosynthesis III^b^	0.07587	0.00201	0.00202	0.00844
Cutin biosynthesis^a, c^	0.00010	0.02782	0.00004	0.02769
Cytokinin-O-glucosides biosynthesis^b^	0.00071	0.01941	0.08622	0.35494
Detoxification of reactive carbonyls in chloroplasts^b^	0.53681	0.00085	0.86982	0.21562
D-mannose degradation^a^	0.00055	0.00482	0.31506	0.11115
D-myo-inositol (1,4,5)-trisphosphate biosynthesis^d^	0.01383	0.02796	0.00549	0.00014
D-myo-inositol-5-phosphate metabolism^d^	0.01089	0.13904	0.04431	0.00233
Dolichyl-diphosphooligosaccharide biosynthesis^b^	0.05277	0.00023	0.62206	0.09771
Folate polyglutamylation^c^	0.99641	0.15204	0.00055	0.02721
Galactose degradation I (Leloir pathway)^c^	0.25818	0.04297	0.00021	0.03955
GDP-mannose biosynthesis^b^	0.00007	0.00298	0.14567	0.05198
Gluconeogenesis III^d^	0.01074	0.05050	0.12302	0.00282
Glucosinolate biosynthesis from dihomomethionine^d^	0.62157	0.44179	0.05763	0.00013
Glucosinolate biosynthesis from hexahomomethionine^d^	0.76899	0.41748	0.12887	0.00012
Glucosinolate biosynthesis from homomethionine^d^	0.88157	0.82380	0.05952	0.00055
Glucosinolate biosynthesis from pentahomomethionine^d^	0.87487	0.55819	0.20668	0.00094
Glucosinolate biosynthesis from tetrahomomethionine^d^	0.87487	0.55819	0.20668	0.00094
Glucosinolate biosynthesis from trihomomethionine^d^	0.87487	0.55819	0.20668	0.00094
Glutathione redox reactions I^a, c, d^	0.00000	0.02432	0.00012	0.00141
Glutathione redox reactions II^a, c^	0.00005	0.03313	0.00007	0.01545
Glycolipid desaturation^b, c^	0.19544	0.00020	0.00028	0.01088
Homogalacturonan biosynthesis^b^	0.00673	0.00046	0.00115	0.01968
Homogalacturonan degradation^a, b^	0.00120	0.00011	0.00314	0.02153
Jasmonic acid biosynthesis^a^	0.00021	0.18112	0.99461	0.04363
L-ascorbate biosynthesis I (L-galactose pathway)^b^	0.00566	0.00146	0.96569	0.14678
Mannitol biosynthesis^a^	0.00053	0.00975	0.75484	0.14698
Pentose phosphate pathway (non-oxidative branch)^c^	0.22936	0.15332	0.00007	0.01277
Phenolic malonylglucosides biosynthesis^c^	0.20109	0.04512	0.00007	0.23758
Phosphatidate metabolism, as a signaling molecule^a^	0.00019	0.01393	0.00578	0.00373
Phospholipid desaturation^b^	0.95288	0.00054	0.22545	0.01008
Phospholipid remodeling (phosphatidylcholine, yeast)^b^	0.38454	0.00299	0.27525	0.04954
Photosynthesis light reactions^a, b^	0.00156	0.00418	0.01072	0.00876
Poly-hydroxy fatty acids biosynthesis^a, b^	0.00001	0.00012	0.04769	0.83503
Reactive oxygen species degradation (mammalian)^a, b, c^	0.00039	0.00307	0.00002	0.04921
Selenate reduction^a, c^	0.00058	0.04222	0.00060	0.00746
Spermine and spermidine degradation III^b^	0.04645	0.00042	0.50380	0.99998
Starch biosynthesis^a, b, d^	0.00000	0.00105	0.05716	0.00000
Starch degradation II^b^	0.06321	0.00000	0.08761	0.08212
Steviol glucoside biosynthesis (rebaudioside A biosynthesis)^d^	0.18468	0.76642	0.00495	0.00068
Sucrose biosynthesis I (from photosynthesis)^d^	0.06807	0.03731	0.00482	0.00054
Sucrose biosynthesis II^b, d^	0.01372	0.00006	0.00124	0.00011
Sulfate reduction II (assimilatory)^a, c^	0.00120	0.06713	0.00004	0.00460
Sulfide oxidation III (sulfur dioxygenase)^a, c^	0.00125	0.55359	0.00008	0.02569
Superpathway of sucrose and starch metabolism I (non- photosynthetic tissue)^c^	0.16930	0.08029	0.00038	0.00329
Trans-zeatin biosynthesis^a^	0.00002	0.00751	0.24322	0.84079
UDP-glucose biosynthesis^c^	0.16930	0.08029	0.00038	0.00329
Very long chain fatty acid biosynthesis I^c^	0.45010	0.13582	0.00008	0.03447
Very long chain fatty acid biosynthesis II^c^	0.96043	0.91563	0.00001	0.26882
Xylan biosynthesis^b, c^	0.01694	0.00319	0.00027	0.00050
Xylogalacturonan biosynthesis^b, c^	0.00288	0.00185	0.00021	0.02373
Xyloglucan biosynthesis^b, c^	0.05061	0.00085	0.00000	0.00769

**Figure 2:**
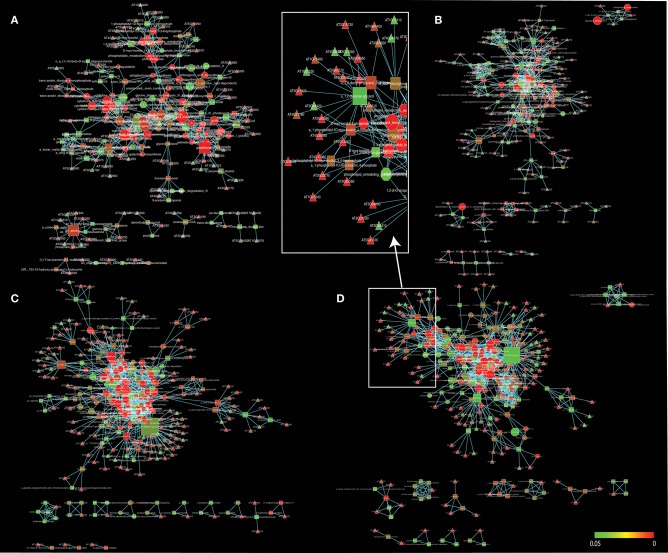
A gene-metabolite-pathway tripartite network of significant changes (*P* ≤ 0.05) for 3 h **(A)**, 6 h (**B**), 12 h (**C**), and 24 h **(D)** cold acclimated *Arabidopsis*. Triangles represent genes, rectangles represent metabolites and octagons represent pathways. Red color range shows most significant genes, metabolites and pathways. Node sizes were scaled to number of neighbors. The networks were visualized by Force directed layout. The white squares represent snapshot of the network. Cytoscape files for network visualization are deposited in https://github.com/gcalab/files.

In our studies, regulated pathways are made explicit through the gene, metabolite and pathway associations of the tripartite gene-metabolite-pathway network that is illustrated in Figure 2. The network and its projections consists of three hierarchical sets of entities (nodes) describing genes (triangles), metabolites (squares), and pathways (octagons), which are connected to each other when significant changes are detected. The size of vertices was scaled to vertex connectivity and colored by *P*-value within the scale 0–0.05 making hub-like behavior and the metabolite-centric approach explicit in the tripartite network.

RPA^m^ led to identification of the most significant effects of cold stress. For example, metabolisms associated with energy generation were significantly detected. The TCA cycle and ethanol degradation were significant pathways which exhibited high network connectivity (see below). Moreover, the glycolytic pathway that is linked to perturbation of energy was significantly identified. On the other hand, ethanol (*P* = 0.01) was a significant metabolite which leads to membrane lipid composition for stabilization (Kaplan et al., 2004). The RPA^m^ identified several common pathways that were significant for all time points, including glutathione redox reactions, sucrose biosynthesis, cellulose biosynthesis, xylan biosynthesis, and D-myo-inositol (1,4,5)-trisphosphate biosynthesis. All of these pathways suggest stress related changes occur in cell membrane lipid composition at all time points through signal metabolism. Moreover, the regulatory role of sucrose in cold acclimation was also reported (Rekarte-Cowie et al., 2008). The following sections describe in detail the most significant effects of cold stress on metabolic networks.

### Carbohydrate and amino acid metabolic activity under cold stress

Plants subjected to cold stress often show water stress symptoms resulting from cold induced water stress (Singh et al., 2012). Adverse environmental stresses including cold trigger rapid regulatory responses of carbohydrate metabolism. For example, starch and sucrose are osmolytes that modulate regulation of water content and cellular dehydration during cold-induced ice formation. While cold stress induces the accumulation of proline, which is a well-known osmo-protectant, we found it was not significantly regulated in our study (*P* = 0.1 for 24 h cold stress). In contrast, sucrose biosynthesis was significantly regulated at all time points, indicating a key role of sucrose in cold perturbation (Table 2).

Carbohydrate metabolic changes were also found associated with the α-D-glucose-1- phosphate (Glc-1-P) metabolite (*P* = 0.02 for 24 h cold acclimation; Figures S2A–**C**), which is an important intermediate in several major carbon fluxes, including those involved in starch, sucrose, and cellulose biosynthesis (Figure 2D). Several carbohydrate degradation pathways were also regulated at different cold acclimation time points, including D-mannose degradation, (1,4)-beta-xylan degradation, sucrose degradation II, galactose degradation I (Leloir pathway), galactose degradation III, and starch degradation II. Taking into account the regulation of these pathways, our results suggest that the breakdown of carbohydrate reserves supported the energetics necessary to survive under cold stress conditions. In previous studies, the whole-plant metabolome of *Arabidopsis* indicated that cold stress increased many metabolites, including amines (asparagine, aspartate, glycine, serine, tryptophan, glutamine and proline, glycine betaine), organic acids (ascorbate, gluconate, malate, and α-ketoglutarate) and carbohydrates (sucrose, maltose, inositol, glucose, fructose, and trehalose) (Cook et al., 2004; Kaplan et al., 2004). In agreement, our method captured glucose, sucrose, maltose, amylopectin, myo-inositol, ascorbate, glycine betaine, L-allo-threonine, L-cysteine, valine, prephenate (precursor of phenylalanine) and other metabolites as significantly regulated (Supplementary File 1, Figure S2). Expectedly, several amino acid metabolic networks were significant in this study, including L-glutamine biosynthesis II, phenylalanine biosynthesis II, tyrosine biosynthesis II, L- glutamine biosynthesis II, and cysteine biosynthesis. Results are consistent with the hypothesis that amino acids and many carbohydrate metabolites were reconfigured under cold stress as a consequence of changes in metabolic activities. The significantly changed metabolites were related to various metabolic pathways including amino acid metabolism, carbohydrate metabolism, cell wall metabolism, fatty acid metabolism, redox metabolism, secondary metabolism, and signal transduction (Figure S2). These cold-induced metabolites may play key roles in signal transduction for gene expression. Therefore, the metabolite-centric approach may reveal whether pathways are hierarchically or metabolically regulated in conjunction with pathway cross-talk (Cakir et al., 2006; Çakir, 2015).

### Energy, protective, and signal metabolic activity under cold stress

**Figure 3:**
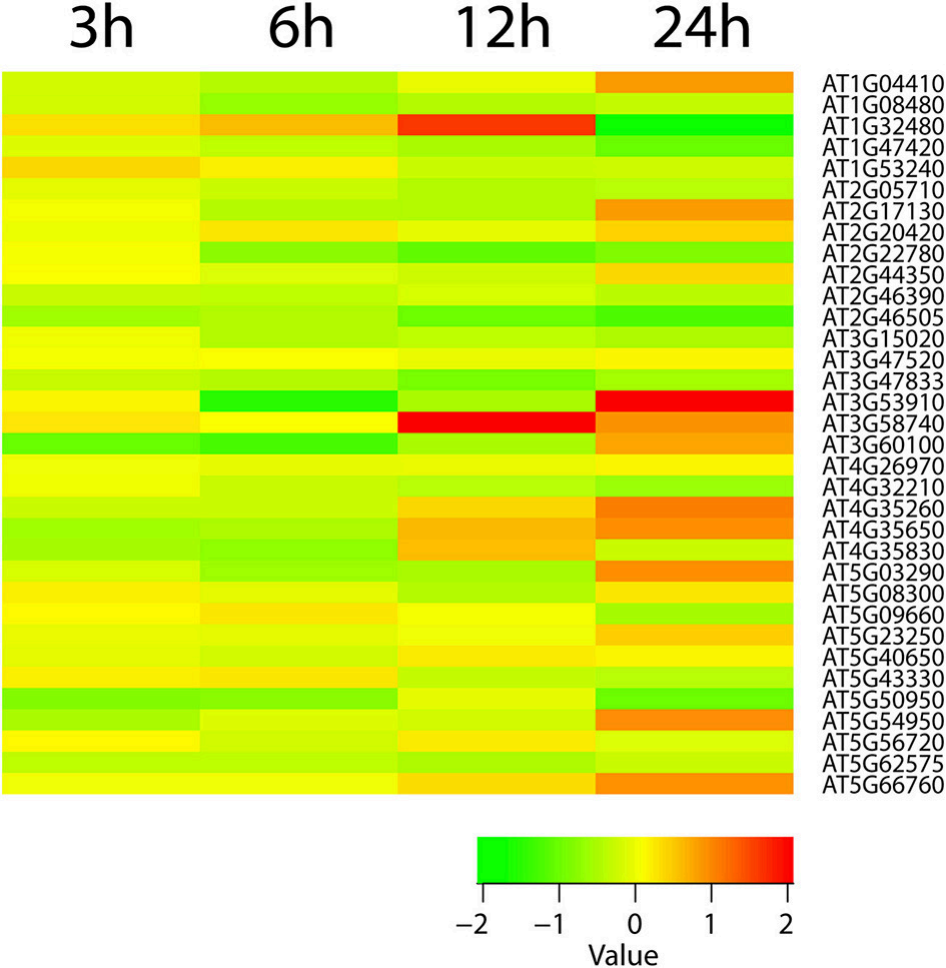
The heatmap diagram of expression profile of TCA cycle genes. Red color hues indicates gene up-regulation while green color hues indicates down-regulation. The green-to-red scale below the heatmap describes expression values.

We identified key pathways that consecutively build protective and signal metabolisms under cold stress. These pathways included callose, ascorbate, D-myo-inositol-5-phosphate, 3-phosphoinositide, xylogalacturonan, xylan, cellulose, homogalacturonan, indole-3-acetyl-amide conjugate biosynthesis, anthocyanidin modification, and phosphatidate metabolism (as a signaling molecule). The 1-phosphatidyl-1D-myo-inositol 4,5-bisphosphate (PIP2) metabolite (*P* = 0.005 for 3 h) is an important signal molecule of the D-myo-inositol-5-phosphate biosynthesis pathway, which is involved in abiotic stress, pollen tube growth, ion channel activity, and guard cell movement (Kost et al., 1999; DeWald et al., 2001; Jung et al., 2002; Liu et al., 2005). Our reporter pathway analysis showed that the gluconeogenesis pathway also scored significantly. This pathway makes glucose from lipids and proteins. Thus, it seems critical to facilitate glucose storage during cold stress when breeding for stress resistance. In addition to providing energy supplements, glucose also plays a key role in membrane stabilization. In addition, the analysis showed glycolysis I, glycolysis II, glycolysis IV, 2-oxoglutarate decarboxylation to succinyl-CoA, pyruvate decarboxylation to acetyl CoA, TCA cycle II (plants and fungi) and, TCA cycle V pathways were significant pathways. In previous studies, TCA cycle intermediates were increased in response to temperature and drought (Kaplan et al., 2004; Urano et al., 2009). When we explored the expression of TCA cycle genes, they were perturbed during early time points of cold stress and then showed only slight increases (Figure 3). Since ATP production needed to maintain adequate ATP/ADP ratios is not decreased in cold stress or cold acclimated *Arabidopsis* (Strand et al., 1997; Hurry et al., 2000), our finding supports the notion that cold stress first induces glycolysis and then adjusts the TCA cycle for ATP production.

**Figure 4:**
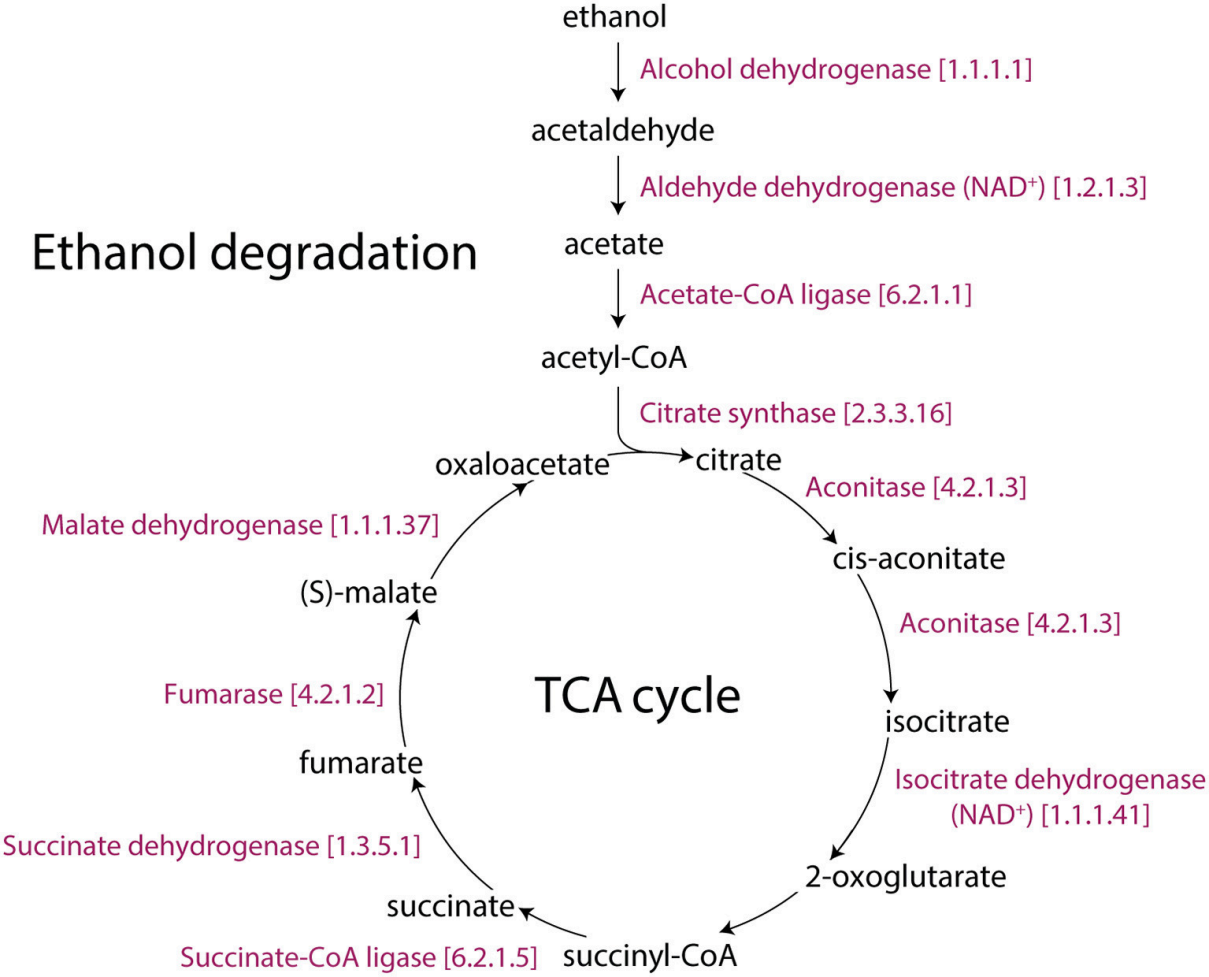
The TCA and ethanol degradation II pathways of *Arabidopsis*. The Aracyc diagram illustrates the enzymes with corresponding EC numbers and substrates that makeup these metabolic routes.

In addition, low-temperature stress causes an accumulation of phosphorylated metabolites (Savitch et al., 2001). This is consistent with ATP production from glycolysis, sucrose degradation, and D-mannose degradation pathways, which support the glycolysis pathway, being also affected in our study. The increase in glycolytic flux requires a coordinated increase in the flux of the pentose phosphate pathway since they are both parallel pathways of the metabolic network that produces ribose 5-phosphate (*P* = 0.017 for 12 h), a precursor for the synthesis of nucleotides. Significantly changed activities of the pentose phosphate (oxidative branch) I and pentose phosphate (non-oxidative branch) pathways are also the source of carbon skeletons for the synthesis of aromatic amino acids, phenylpropanoids, and their derivatives (Herrmann and Weaver, 1999). AT1G20950, AT4G04040, AT1G76550, and AT1G12000 encode fructose-6- phosphate 1-phosphotransferase (EC 2.7.1.90). This enzyme catalyzes the initial reaction of the glycolysis IV (plant cytosol) pathway, which converts β-D-fructofuranose 6-phosphate into fructose-1,6-bisphosphate. In the last reaction of glycolysis IV, AT3G22960, AT5G52920 and, AT3G55650 (pyruvate kinase) encode for enzymes that catalyze the conversion of phosphoenolpyruvate into pyruvate. AT2G29350 encodes the alcohol dehydrogenase (EC 1.1.1.1) enzyme that converts ethanol into acetaldehyde. In turn, AT3G48000, AT1G44170, and AT3G24503 (aldehyde dehydrogenase; EC 1.2.1.3) catalyze the conversion of acetaldehyde into acetate, and AT5G36880, AT3G16910 and, AT1G55320 (acetate-CoA ligase; EC 6.2.1.1) catalyze the conversion of acetate into acetyl-CoA. Previous studies have reported a pyruvate dehydrogenase bypass that produces ethanol to support the TCA cycle and lipid biosynthesis (Mellema et al., 2002; Gass et al., 2005; Yue et al., 2014). Additionally, the antimycin A inhibition of the mitochondrial electron transport chain triggered the immediate production of ethanol and a fast re-arrangement of metabolic pathways. Ethanol availability and its fermentation compensated for reduced ATP production (Obermeyer et al., 2013). Our study shows that glycolysis and ethanol fermentation (*P* = 0.01 for 6 h, Table 1, Figure S2B) under cold stress diverted energy into the TCA cycle, turning it into an alternative energy producing pathway of *Arabidopsis* (Figure 4).

**Figure 5:**
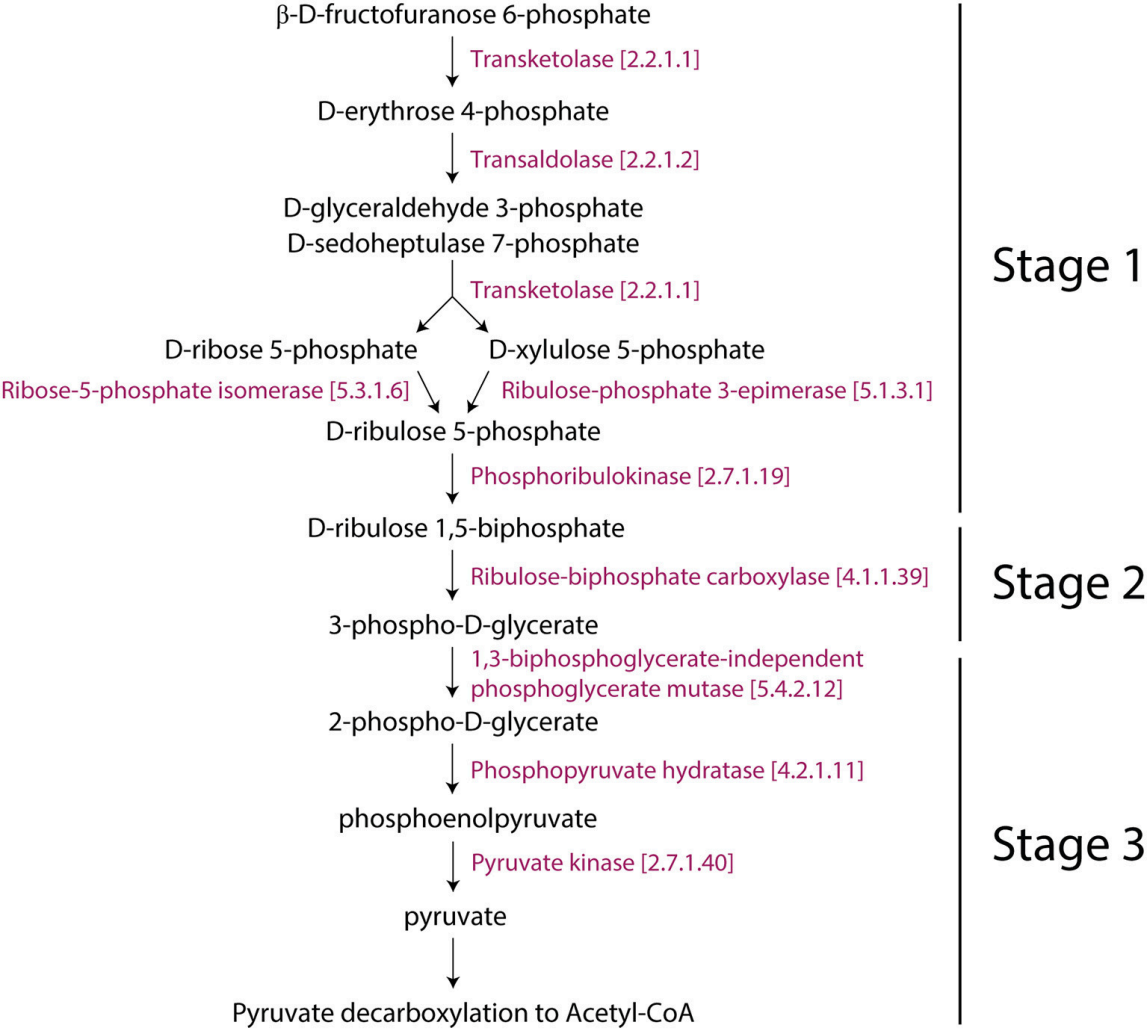
Representation of the Rubisco shunt in *Arabidopsi*s with consecutive enzymes. The pathway was taken from Aracyc.

Interestingly, the Rubisco shunt was also significantly changed in *Arabidopsis*. In general, the glycolysis I (from glucose 6-phosphate) and glycolysis IV (plant cytosol) pathways facilitate the conversion of carbohydrates to oil. Pyruvate, which is the end product of glycolysis, is further decarboxylated into acetyl-CoA, which is a precursor for fatty acid and oil biosynthesis (Neuhaus and Emes, 2000). However, this process causes the loss of one-third of carbon as CO_2_ (Schwender et al., 2004). This is not metabolically advantageous. When compared to glycolysis, the Rubisco shunt increases the efficiency of carbon conversion, producing 20% more acetyl-CoA with 40% less carbon loss. Rubisco operates a metabolic route between carbohydrate and lipid metabolism in coordination with Acetyl-CoA derivatives and CO_2_ producing pathways (2-oxoglutarate decarboxylation to succinyl-CoA and pyruvate decarboxylation to acetyl-CoA), which were reprogrammed for cold acclimation. This occurs in three stages: (1) the conversion of hexose phosphates to ribulose-1,5-bisphosphate by phosphoribulokinase (the non-oxidative reaction); (2) the conversion of ribulose-1,5-bisphosphate and CO_2_ (mostly produced by PDH3) to PGA by Rubisco (ribulose 1,5-bisphosphate carboxylase/oxygenase); and (3) the metabolism of PGA to pyruvate and then to fatty acids (Figure 5). We hypothesized that the Rubisco shunt balances carbohydrate and oil production in order to make efficient use of the carbon pool under cold stress.

### Lipid metabolic activity under cold stress

Several fatty acid pathways were impacted by cold stress, including very long chain fatty acid biosynthesis I and II, choline biosynthesis III, triacylglycerol biosynthesis, diacylglycerol biosynthesis (polyunsaturated fatty acids), poly-hydroxy fatty acids biosynthesis, phospholipid remodeling, phospholipid desaturation, glycolipid desaturation, phospholipases, and phosphatidylcholine biosynthesis I and II. Cold, heat, and osmotic stress change the physical properties of lipids in cell membranes (Los and Murata, 2004). It is well-documented that the cell membranes are a primary site of cold-induced damage (Thomashow, 2001; Matteucci et al., 2011). In these studies, cold acclimation activates mechanisms that protect the fluidity of membranes by ensuring that associated enzymes have optimal activity. Cold responsive plant species can change membrane lipid fluidity by increasing the levels of unsaturated fatty acids (Matteucci et al., 2011). These molecules are crucial to cold tolerance and photosynthesis (Meyerowitz and Somerville, 1994). In one study, profiling membrane lipids of *Arabidopsis* subjected to 3 days of cold acclimation revealed an increase in desaturation in all measured phospholipids (Welti et al., 2002). Similarly, low temperature resulted in an increase in the degree of fatty acid unsaturation in chickpea (Bakht et al., 2006, p. 2). In accordance with these lipid profiling studies, our reporter pathway analysis showed changes of fatty acid metabolism necessary to alter membrane composition in *Arabidopsis* under cold stress.

### Cell wall metabolism under cold stress

Exposure to chilling temperature causes alterations in the composition of the cell wall and in the activities of cell wall-modifying enzymes (Le Gall et al., 2015). For example, cell wall strength was increased in grape suspension culture and apple under cold acclimation (Rajashekar and Lafta, 1996). We identified several pathways related to cell wall composition as significantly changed. Among them, cellulose biosynthesis, xylan biosynthesis, homogalacturonan biosynthesis, xylogalacturonan biosynthesis, xyloglucan biosynthesis, cuticular wax biosynthesis, cutin biosynthesis were significant. Xylan biosynthesis comprises the synthesis of several components of the cell wall such as xylan and arabinoxylan. In addition, homogalacturonan (*P* < 0.05 for 3, 6, and 12 h) accounts for 60% of the total pectin in primary cell walls of plants (Mohnen et al., 1996). Its content was reported to increase in oilseed rape leaves in response to cold exposure (Solecka et al., 2008). Thus, pectin (Table 1, Figures S2A,D), which was a significant metabolite of our study, appears a key cell wall component affected by the plant response to cold stress.

### Methanol production under cold stress

We found that pectin-interacting methanol was a crucial metabolite in the 3 and 24 h cold treatments (Figures S2A,D). Most plants produce and extrude methanol, especially because of pectin demethylation during the early stages of leaf expansion (Fall and Benson, 1996). The degree of methylation plays a key role in properties of the cell wall and cell adhesion (Liu et al., 2014). PEG treatment decreased the degree of methylation of pectin in root tips of *Phaseolus vulgaris*. This metabolite is incorporated into the methyl groups of numerous metabolites in higher plants, including methionine, serine and phosphatidylcholine. Additionally, methanol induces the de novo synthesis of methyl-β-d-glucopyranoside (Gout et al., 2000). In this regard, overwintering leaves of alpine Rosaceae plant *Dryas octopetala* produced the highest levels of methyl-β-d-glucopyranoside (Aubert, 2004). Therefore, methanol appears involved in cold stress and its role merits further study.

### Redox metabolism under cold stress

Our reporter pathway analysis identified several redox metabolisms, including L-ascorbate biosynthesis I (L-galactose pathway), L-ascorbate biosynthesis V, ascorbate glutathione cycle, glutathione redox reactions I and II, gamma-glutamyl cycle, formaldehyde oxidation II (glutathione-dependent), sulfate reduction II, formaldehyde oxidation II and thioredoxin pathway in cold stress (Supplementary File 1, Figure 2). L-ascorbic acid, popularly known as vitamin C, is one of the most abundant water-soluble antioxidants. Its ability to scavenge free radicals and ROS produced by drought, salt, and high or low temperature stress in plants demonstrates its important role in plant growth, development and abiotic stress tolerance (Zhang et al., 2015). Additionally, we found that glutathione (*P* < 0.05 for 3, 12, and 24 h) was a key metabolite in redox metabolism (Figure S2). When coupled with the ascorbate/glutathione cycle and the redox system, ascorbate can effectively regulate cellular hydrogen peroxide levels in plants (Ishikawa et al., 2008). Most genes encoding enzymes in this pathway, including GDP-mannose pyrophosphorylase (GMP) (Badejo et al., 2008), GDP-mannose-3′,5′-epimerase (Zhang et al., 2011), GDP-L-galactose phosphorylase (Bulley et al., 2012), L-galactose-1-phosphate phosphatase (Zhou et al., 2012), L-galactose dehydrogenase (Gatzek et al., 2002), and L-galactono-1,4-lactone dehydrogenase (Liu et al., 2011), have been shown to modulate ascorbic acid levels in plants. Ascorbate directly counteracts ROS and participates in the regeneration of α-tocopherol, which scavenges both ROS and lipid peroxyl radicals and zeaxanthin, which plays roles in the photoprotective xanthophyll cycle (Bielen et al., 2013). The glutathione redox pathway maintains the reduction of various molecules such as peroxides, glutaredoxin, or protein-disulfides that are formed from two molecules of glutathione (GSH) and are catalyzed into the oxidized form of glutathione disulfide (Milla et al., 2003). A recent study showed that the accumulation of both reduced and oxidized ascorbate coupled with oxidized and reduced glutathione were induced by cold and salt stresses (Wang et al., 2016). We also identified zeaxanthin (*P* = 0.04) and lutein metabolism (carotenoid metabolisms; *P* = 0.02), functioning as antioxidants. Similarly, *Coffea canephora* showed higher contents of the zeaxanthin and lutein in cold perturbation (Partelli et al., 2009). Aerobic respiration III (alternative oxidase pathway) was found regulated in cold stress. This pathway is not associated with ATP production. Nevertheless, alternative oxidase activity in this pathway decreases the formation of ROS by hindering the over-reduction of the ETC (Møller, 2001). Therefore, pathways linked to redox metabolism are expected to be active under cold stress for rebalancing the impaired redox state of the cellular machinery.

### Photosynthesis pathway under cold stress

Photosynthesis is undoubtedly a crucial sensor of stress in plants. Photosystem I (PS I) and II (PS II) and Rubisco are considered to act as primary stress sensors of the chloroplast (Koç et al., 2015c). Stress perturbation of these sensors generate signals such as ROS production, change of sugar levels, redox reactions of the photosynthetic electron transport system, and energy imbalance, which cause metabolic/molecular reconfiguration of stress adaptation (Biswal et al., 2011; Koç et al., 2015b). Abiotic stresses can also affect the rates of primary photochemical reactions and reduce the activities of the enzymes of the Calvin cycle (Biswal et al., 2011). We identified that the Calvin-Benson-Bassham cycle (Calvin cycle) and photosynthesis light reactions were significantly regulated in *Arabidopsis* during cold acclimation. The Calvin cycle determines the net fixation of CO_2_ in plants. Collectively, the reporter metabolites of the Calvin cycle and Rubisco shunt indicate that cold reduces the efficiency of carbon fixation. However, Rubisco participates in a previously described metabolic route between carbohydrate and lipid metabolism mediated by increases of acetyl-CoA levels. This strategy developed by plants can be thought as efficient metabolic flux to carbon storage (Schwender et al., 2004). It is well known that plants reduce the oxidative damage caused by low temperatures by down-regulating light reactions known to increase the oxidative damage. In this respect, we also identified that the chlorophyll a degradation I pathway was regulated under cold acclimation. Several studies reported that cold stress causes degradation of chlorophyll (Tewari and Tripathy, 1998; Liu et al., 2012; Singh et al., 2012). To this end, the chlorophyll biosynthetic pathway, which plays significant roles in photosynthesis and in avoiding the accumulation of phototoxic chlorophyll intermediates, must be tightly regulated (op den Camp et al., 2003; Rodríguez et al., 2013).

### Hormone metabolic activity under cold stress

Increasing evidence indicates that synergistic or antagonistic hormone cross-talk play key roles during abiotic stress (Santner and Estelle, 2009). Plant hormones often regulate metabolism by rapidly modulating transcriptional factor activity (Peleg and Blumwald, 2011). Our reporter pathway analysis identified phytohormone metabolism of auxin (IAA) biosynthesis VII, indole- 3-acetyl-amide conjugate biosynthesis, ethylene biosynthesis I, cytokinins 7-N-glucoside biosynthesis, and cytokinin-*O*-glucosides biosynthesis as significantly changed during cold acclimation (Figure 2). Auxin is involved in many physiological processes, including plant growth, development and the abiotic stress response (Jain and Khurana, 2009). In *Arabidopsis*, cold stress is known to affect the auxin transport pathway by inhibiting various intracellular proteins, including auxin efflux carriers (Shibasaki et al., 2009). Similarly, the expression profile of the ARF and Aux/IAA gene family members of *Arabidopsis* changed during cold acclimation (Hannah et al., 2005). Transcriptome analysis in rice showed that over 154 genes were induced and 50 were repressed by auxin under desiccation, salt, and cold stress (Jain and Khurana, 2009). Most IAAs are considered to be conjugated to a number of amino acids (Staswick et al., 2005). Some of these amino acid modifications can be reversed through the activity of amido hydrolases (Davies et al., 1999), suggesting that compounds such as IAA-alanine and IAA-leucine are storage forms of auxin that can be further metabolized to feed into the free auxin pool (Staswick et al., 2005). Indole-3-butyrate (IBA) was also a significantly changed metabolite (*P* = 0.01 for 24 h cold stress), suggesting it functions as an endogenous IAA precursor (Ljung, 2013). Some investigations of IBA biosynthesis show that its level is likely regulated by plant hormones and various stresses (Ludwig-Müller et al., 1995, 1997). Another phytohormone, ethylene, regulates germination, fruit ripening, organ abscission, and pathogen, senescence, and biotic/abiotic stresses (Chen et al., 2005). Increased ethylene levels confer enhanced freezing tolerance in *Arabidopsis* by decreasing freezing tolerance and inhibiting ethylene biosynthesis/signaling (Shi et al., 2012). Ethylene synthesis is sustained by inhibiting 1-aminocyclopropane-1-carboxylate oxidase (Vriezen et al., 1999). Since AT1G12010 and AT1G05010 (1-aminocyclopropane-1-carboxylate oxidase), which convert 1-aminocyclopropane-1-carboxylate to ethylene, were down-regulated, we find evidence that the ethylene pathway was perturbed under cold stress (Figure S3).

The signaling and response to cold stress has been associated with cytokinin signal transduction and a subset of a two-component signaling (TCS) system (Jeon et al., 2010; Jeon and Kim, 2012). The ARABIDOPSIS RESPONSE REGULATOR1 mediates signaling through the activity of AHP2, AHP3, or AHP5 to induce a subset of type-A ARRs (Jeon et al., 2010; Jeon and Kim, 2012). Together with cytokinin, this regulates tolerance and the cold stress response. However, the exact role of cytokinins on the cold stress response is unclear and needs to be further explored. In addition, our reporter metabolite analysis also identified some other phytohormone-related pathways of brassinosteroid (superpathway of C28 brassinosteroid biosynthesis), jasmonate (jasmonic acid biosynthesis), and gibberellin (GA12 biosynthesis) hormones (Table 2, Supplementary File 1). Our findings suggest plant hormones may coordinate responses to increase the plant adaptability to cold stress.

### The structure of tripartite networks

Stress responses have been widely studied in plants. Here we examine stress-induced patterns by visualizing changes in metabolites and pathways using reporter metabolite analysis in absence of metabolome data. The tripartite networks quantify the dynamic aspects of stress-induced change by making connectivity and modularity explicit in the network structures that describe the time resolved cold response. All tripartite networks resulted in disconnected undirected graphs. Only few small intra-connected components were detected to be isolated from the large connected component. As time progresses, the appearance of nodes and links emerge in the tripartite networks with increasing cold acclimation. The emergent networks had 218, 246, 306, and 320 nodes with a network density (actual/possible number of edges) of 0.023, 0.021, 0.026, and 0.028 at 3, 6, 12, and 24 h of cold acclimation, respectively (Table 3). An analysis of network topology showed that networks had diameters of 12, 9, 10, and 9, respectively, and exhibited average numbers of neighboring nodes of 5.1, 4.3, 3.9, and 3.7, respectively (Table 3). These results suggest that tripartite networks become more compact and gain decentralized infrastructure with increasing cold acclimation.

**Table 3:** Graph properties of the tripartite networks at different times of cold acclimation.

	**3h**	**6h**	**12h**	**24h**
Density	0.023	0.021	0.026	0.028
Average clustering coefficient	0.69	0.65	0.75	0.77
Diameter	12	9	10	9
Total nodes	218	246	306	320
Exclusive nodes	125	145	193	196
Total edges	545	640	1192	1404
Exclusive edges	319	367	764	962
Average path length	5.109	4.337	3.898	3.696
KS fit statistic	0.46	0.45	0.40	0.45
*P*-value	0.000003	0.000001	0	0
Gamma (-γ)	1.37	1.39	1.12	1.07
Log-likelihood	−16.50	−17.47	−30.51	−37.86
Coefficient determination (*R*^2^)	0.78	0.83	0.77	0.69

**Figure 6:**
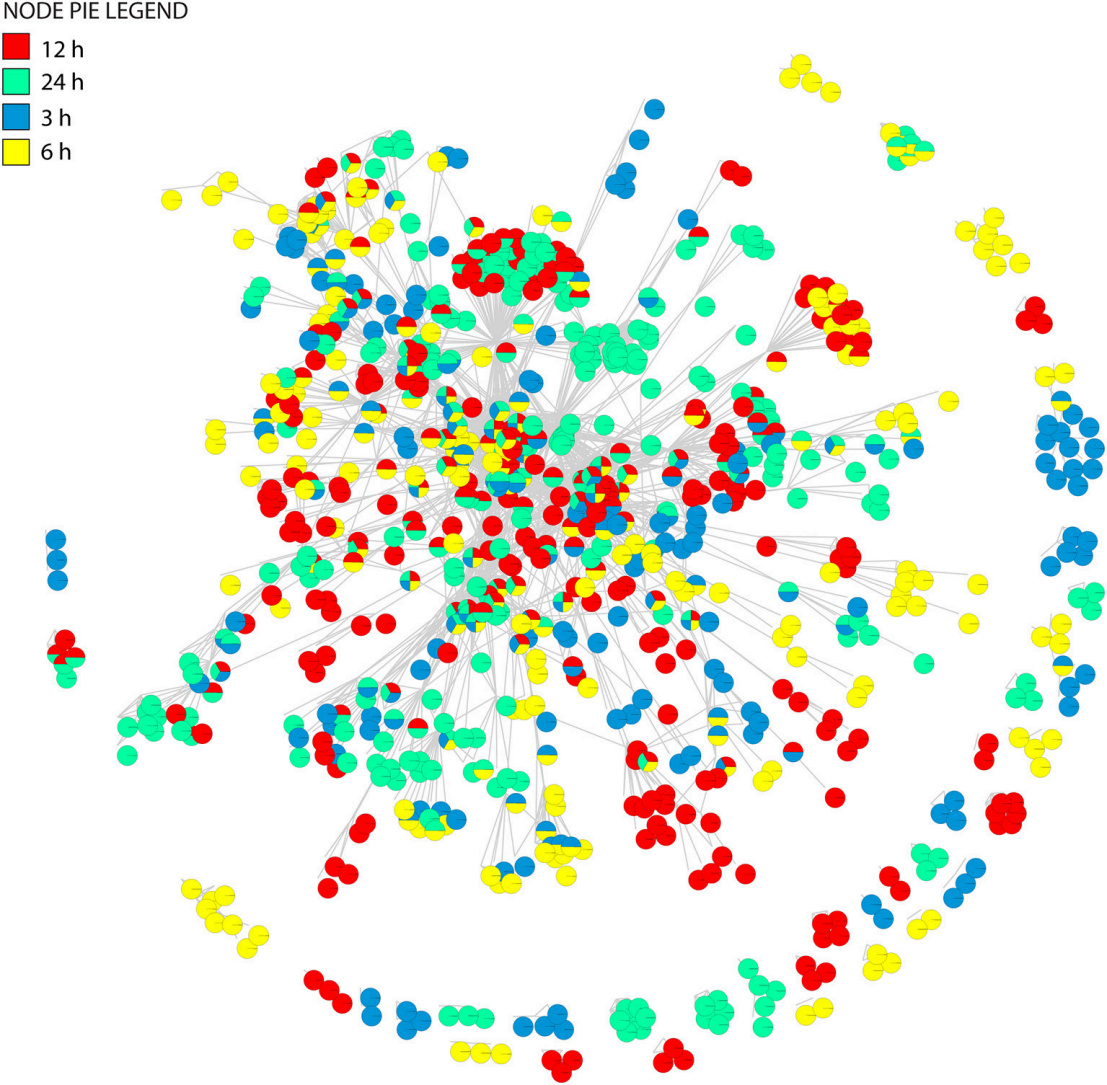
The union of tripartite networks. The pie-node representation enables to identify presence/absence of individual nodes across four time dependent networks.

**Figure 7:**
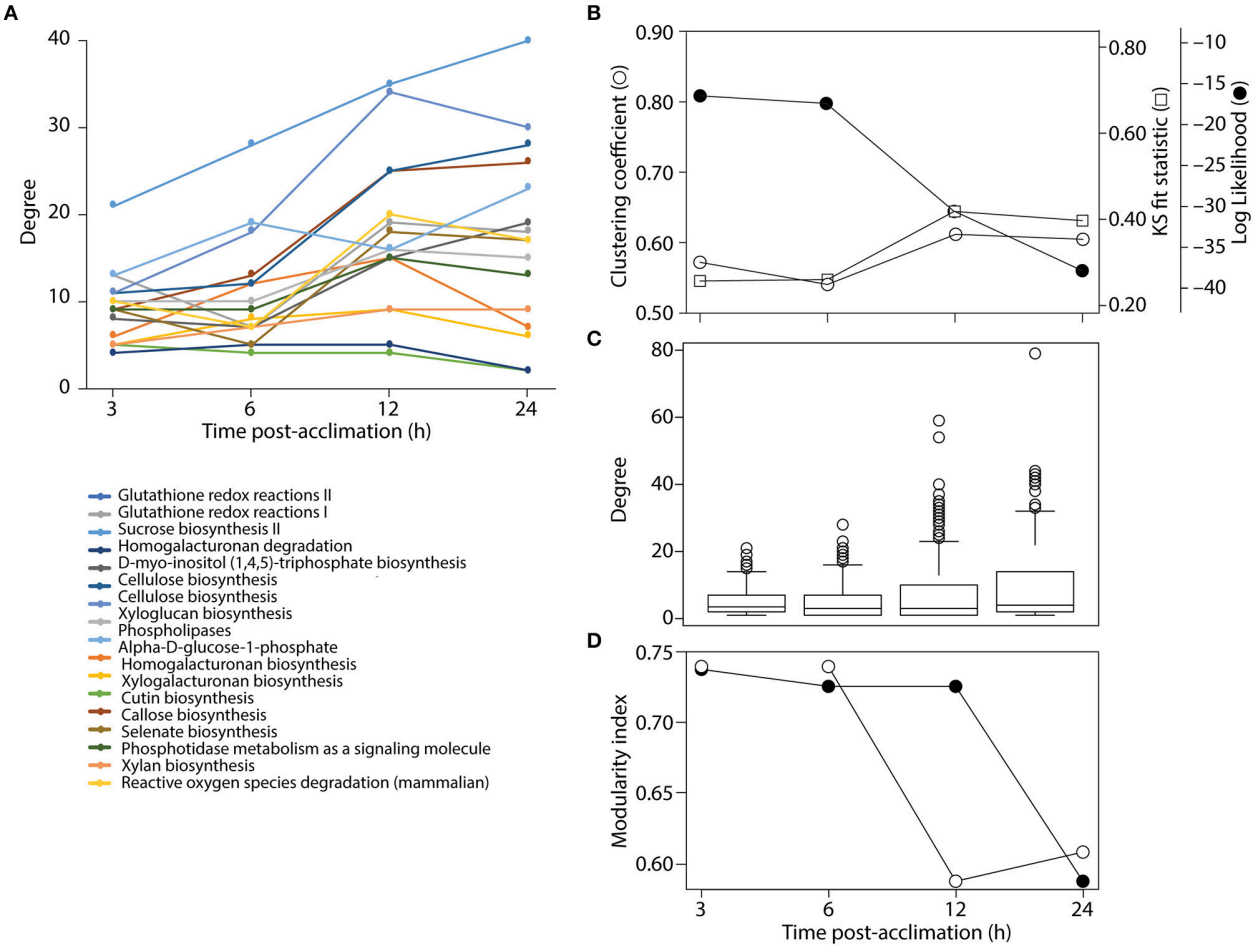
Analysis of network structure. **(A**) Connectivity of common nodes for all time points. **(B)** Analysis of network modularity using the clustering algorithm and some statistical descriptor of power law behavior of tripartite networks over time. (**C**) Boxplots show the span of connectivity measured by node degree for each time point. (**D**) Analysis of modularity with the FGC (closed circles) and NG (open circles) algorithms.

Clustering analysis revealed that networks were well-structured. We see more connected rewiring patterns in response to stress over time (Figures 2, 6). Remarkably, the nodes that are common between all the time points of cold treatment (centrally located in Figure 6) exhibit an increasing connectivity trend that reaches a plateau at 12 h of cold acclimation (Figure 7A). For example, sucrose biosynthesis II showed the most significant pattern of increase, suggesting that these networks are highly dynamic in their ability to rewire over time of cold acclimation. When we compared the four time points by overlaying networks on each other, patterns of connectivity over the course of stress were visible, including the existence of mutually exclusive nodes and edges that decompose graphs into maximal non-separable subgraph blocks that are nearly non-overlappable. The number of exclusive nodes increased with time but reached a plateau at 12 h of acclimation. Similarly, the number of exclusive edges increased with time throughout the timeline (Table 3). These trends fit patterns of network clustering that suggest the network is modular (see below).

The connectivity of a network can be characterized by the probability *P(k)* that a node has *k* connections. For inhomogeneous networks, highly connected nodes get more connections. In these random networks, the probability *P(k)* of a node being connected to *k* other nodes decays as a power law, *P(k)* ~ *k*^−γ^, without a characteristic scale (Jeong et al., 2000). For example, metabolic networks have a tendency to follow scale free network behavior with γ = 2.1–2.4 exponents (Jeong et al., 2000). In order to test if the tripartite networks at different times of cold acclimation tend to follow the scale-free distribution we measured power law behavior with network statistics (Table 3). We plotted log *P(k)* vs. log *k* for all tripartite networks (Figure S4). Log-log plots showed a limited correlation fit [Kolmogorov–Smirnov (KS) fit ranging 0.4–0.46] to power law behavior (γ ranging from 1.06 to 1.37). The γ for the tripartite networks is lower than the γ reported for metabolic networks (Jeong et al., 2000). Determination coefficients (*R*^2^) of ~ 70% support the linear model. KS fit statistic higher than >0.10 and low *P*-values of the KS test (< 0.05) (Clauset et al., 2009) rejected network data being drawn from the fitted power-law distribution for all cold acclimation time points. Similarly, the log-likelihoods of the fitted power law parameters were much lower than zero for all networks. This suggests the power law distribution is unlikely. Overall, the statistical analysis of tripartite networks over time rejected power law behavior (Figure 7B, Table 3). This reveals that tripartite networks were not dictated by hubs. Instead they harbored large components of highly rewired vertices.

Boxplot graphs show connectivity variation measured by the number of incident connections of a node (its degree) (Figure 7C). Variation was minimal at 3 h cold acclimation and maximal at 24 h. Results explicitly show that stress propels the dynamic behavior of tripartite networks. Networks can show modular behavior. Modularity can be estimated with the average clustering coefficient (*C*), which describes the ratio of connected triplets in the graph normalized over all vertices and ignoring the direction and weights of the edges (Aziz et al., 2016). From a topological perspective, the tripartite networks of *Arabidopsis* have high clustering coefficients, with values that increased from 0.69 to 0.77 along the cold acclimation sequence (Table 3). This property is suggestive of modular organization. This trend corresponds to the KS fit statistics which in turn reject power law behavior. The modularity of tripartite networks was assessed using the FGC and NG algorithms that reveals scores of communities in the networks. In particular, FGC provides a hierarchical perspective of agglomerative community structure. Positive index values indicate the significant existence of modules in the connectivity structure of the network. Notably, FGC and NG similarly showed high modularity upon stress application (Figure 7D). Indices decreased at 12 and 24 h post-acclimation but remained positive. Analysis of modularity shows that connectivity increases (exclusive nodes and edges, densely interconnected functional modules) along with high cluster coefficients (Figures 6, 7, Table 3). High indices indicate that the connectivity of vertices belonging to modules is higher than those between modules. In turn, low indices indicate high community structure. High modularity appears in the networks as *Arabidopsis* progressed toward new programming of metabolic diversity and increased modularity upon perturbation. This suggests that tripartite networks under cold stress are characterized by a high intrinsic potential modularity. Apparently, this pattern was dictated by a development of network clusters within modules outside the context of scale free organization. Interestingly, shared vertices (commonly present in four networks) were highly connected and located in the center of the network while individual vertices were located toward the periphery (Figure 6). Among the common pathways for all time points, carbohydrate, lipid, cellulose, signal and redox metabolisms were explicit. This observation supports stress-induced trends toward redox metabolism, signal metabolism, carbon metabolism and cell wall metabolism (Supplementary File 2). This feature also uncovers the rewiring change of network with stable components of physiology. On the other hand, we see dynamic change of metabolites and pathways as a response to stress application, which evolves modular structure of the tripartite networks.

### Network cluster analysis

**Figure 8:**
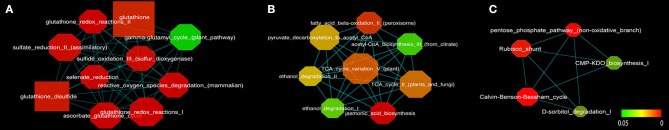
Sub-network association of pathways at selected time points. (**A**) Sub-network of redox metabolism at 3 h cold acclimation. (**B**) Sub-network of energy metabolism at 3 h cold acclimation. (**C**) Sub-network of carbon metabolism at 12 h cold acclimation. Red color range shows most significant genes, metabolites, and pathways.

In addition to modules, extracting clusters can identify highly connected subgraphs in which nodes have more interactions than the rest. The association between components of a cluster can reduce network complexity. We used cluster analysis to highlight key metabolic pathways and delineate central associations between pathways. Using cluster analysis, we identified >20 clusters of sizes from 3 to 27 (data not shown). For example, extraction of a sub-network from the 3 h cold acclimation network showed redox metabolisms linked to each other (Figure 8A). Interestingly, the jasmonic acid pathway was highly connected to the TCA cycle, acetyl–CoA biosynthesis, pyruvate decarboxylation to acetyl-CoA, and ethanol degradation pathways (Figure 8B). Jasmonate targets the mitochondria of cancer cells by perturbing them (Rotem et al., 2005). Similarly, jasmonate may induce perturbation of mitochondrial function in plants under stress conditions. It would be interesting to validate this hypothesis in future studies. Furthermore, plants seem to increase the glycolysis and ethanol degradation pathways under cold stress since the bio-energetic machinery is impaired. The resulting sub-network again supported the notion that the TCA cycle works alongside ethanol degradation to produce excess of acetyl-CoA. Focusing on the Rubisco shunt and Calvin cycle interaction, we extracted a sub-network from the 12 h cold acclimation network. The resulting sub-network also showed main carbon metabolic pathways were highly connected to each other (Figure 8C). Plants confront many imbalances in carbon metabolism under perturbation. As expected, photosynthesis is significantly down-regulated whereas starch, sucrose and amino acid metabolisms are up-regulated in such stress conditions. This suggested that when photosynthesis is perturbed, plants may increase the activity of alternative pathways to modulate CO_2_ fixation, including activating the Rubisco shunt. This involves maintaining a steady-state of energy metabolism (Figure 8C).

## Conclusion

A metabolite-centric reporter pathway analysis of transcriptomes uncovered a number of metabolites and pathways that differentially modulate the plant response to cold stress in *Arabidopsis*. Notably, cold stress levies great demands on energy production and biochemical requirements. Initially, plants mobilize glycolysis to rapidly produce ATP and divert the main carbon flux routes to amino acid and lipid metabolic pathways. In this process, many metabolic pathways are activated to produce cellular materials needed to sustain stress. We find that energy rich metabolites, such as ethanol, may help the functioning of the TCA cycle under perturbation. Signal pathways and structural integrity related pathways were also activated. Clearly, our metabolite-centric reporter pathway analysis provides a new framework with which to help scientists achieve a deeper understanding of the co-regulation of metabolites and metabolic pathways. Our analysis also revealed modularity and rejection of the power law behavior in the structure of tripartite networks of gene-metabolite-pathway connectivity.

## Author contributions

IK and IY performed experiments. IK, IY, and GC-A analyzed the data. IK and GC-A wrote the manuscript.

### Conflict of interest statement

The authors declare that the research was conducted in the absence of any commercial or financial relationships that could be construed as a potential conflict of interest.

## Supplementary material

Figure S1 Principal component analysis (PCA) of control and cold acclimated samples derived from all genes from the Affymetrix ATH1 microarray chip **(A)** and the subset of metabolic genes **(B)**.

Figure S2 Subgraph of tripartite network showing significantly changed metabolites (*P* ≤ 0.05) for 3 h **(A)**, 6 h **(B)**, 12 h **(C)**, and 24 h **(D)** cold acclimated *Arabidopsis*. Nodes represent metabolites. Their sizes were scaled to number of neighbors.

Figure S3 Mapping gene expression onto the ethylene pathway of the KEGG database (red circle). The graph was produced with the R pathview library. Rectangles represent enzyme-encoding genes labeled with Enzyme Commission (EC) number. Red color hues indicate gene up-regulation while green color hues indicate down-regulation. EC:1.14.17.4, aminocyclopropanecarboxylate oxidase.

Figure S4 Log-log plots show a scatter plot of natural log of *k* against *P(k)* of the tripartite networks for each time point.

## Abbreviations

RPA^m^: Metabolic centric reporter pathway analysis.
