# Supplementary figures and images for: Metabolite-Centric Reporter Pathway and Tripartite Network Analysis of Arabidopsis Under Cold Stress
Source: Front Bioeng Biotechnol. 2018 Sep 11;6:121. doi: 10.3389/fbioe.2018.00121 (PMC6143811; doi:10.3389/fbioe.2018.00121)

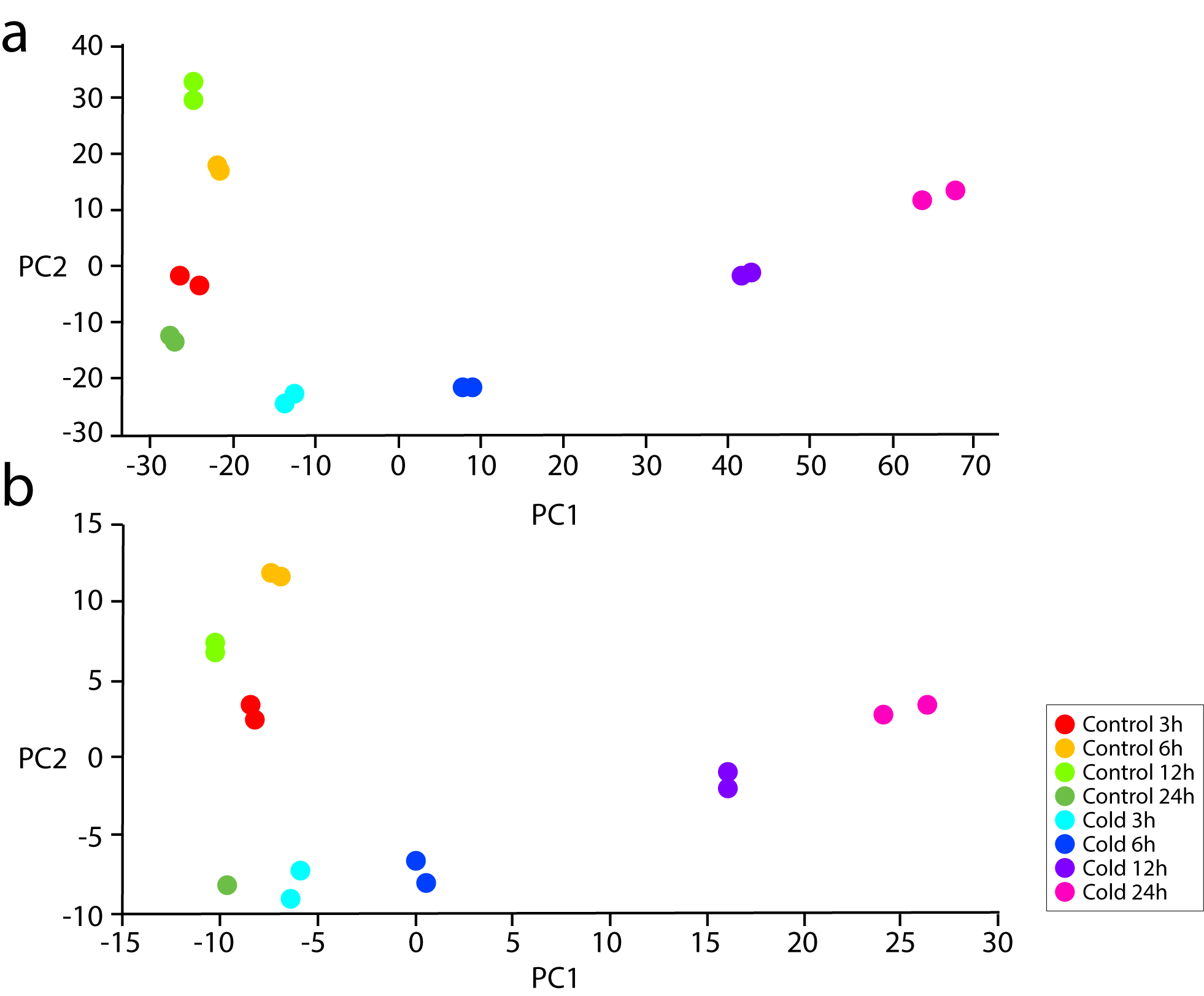

Supplement: Figure S1 — Principal component analysis (PCA) of control and cold acclimated samples derived from all genes from the Affymetrix ATH1 microarray chip (A) and the subset of metabolic genes (B). [file Image_1.TIF]

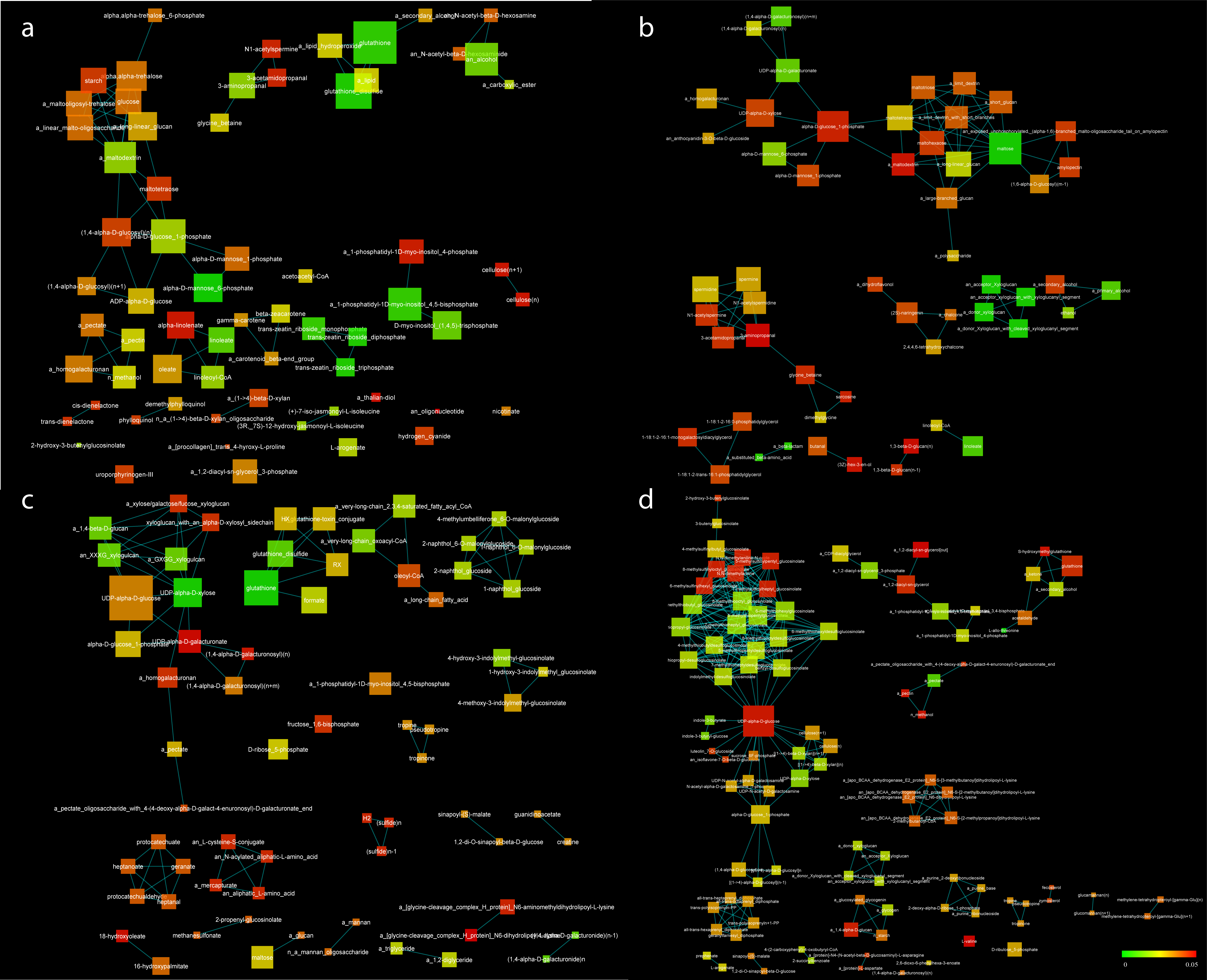

Supplement: Figure S2 — Subgraph of tripartite network showing significantly changed metabolites (P ≤ 0.05) for 3 h (A), 6 h (B), 12 h (C), and 24 h (D) cold acclimated Arabidopsis. Nodes represent metabolites. Their sizes were scaled to number of neighbors. [file Image_2.TIF]

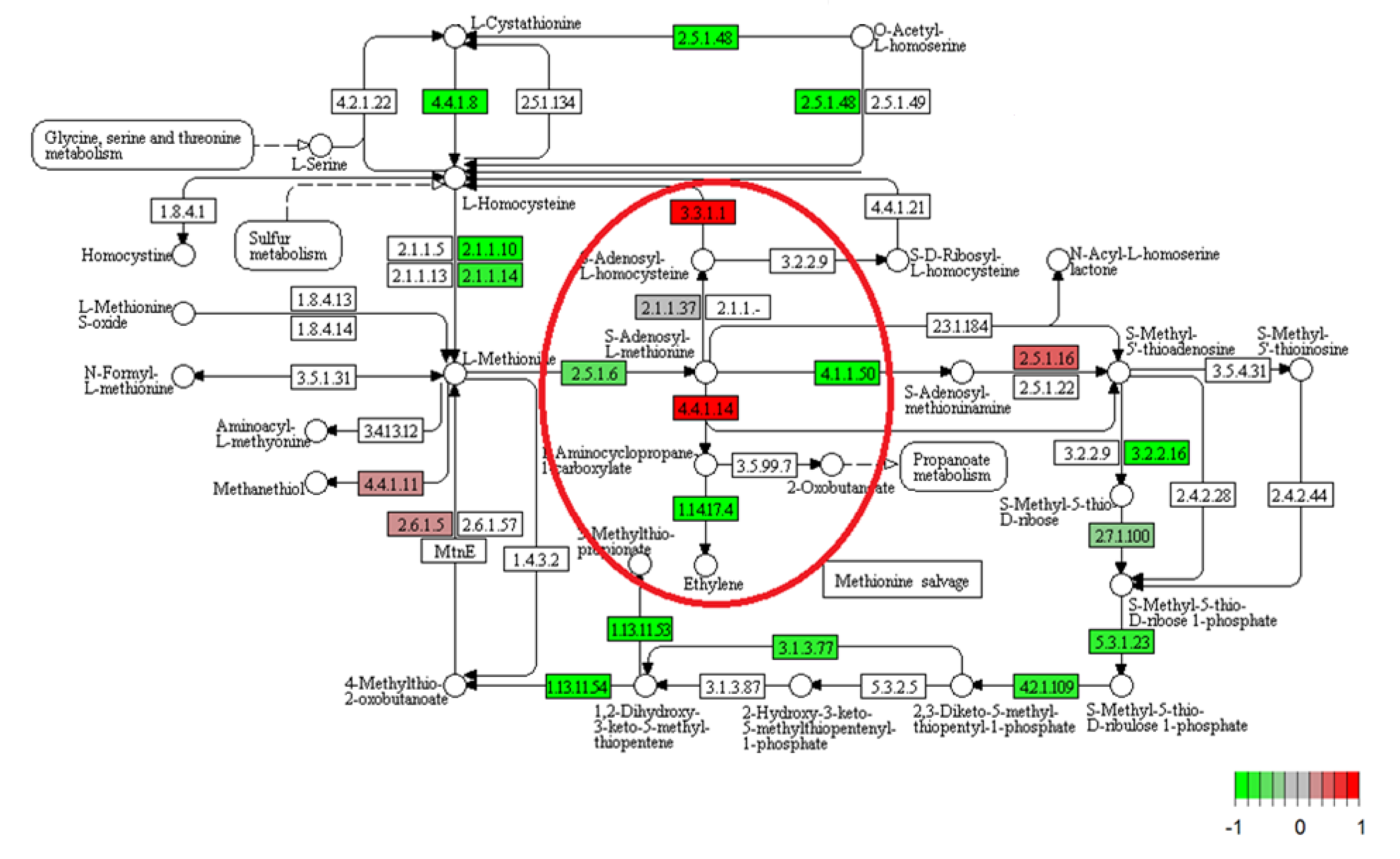

Supplement: Figure S3 — Mapping gene expression onto the ethylene pathway of the KEGG database (red circle). The graph was produced with the R pathview library. Rectangles represent enzyme-encoding genes labeled with Enzyme Commission (EC) number. Red color hues indicate gene up-regulation while green color hues indicate down-regulation. EC:1.14.17.4, aminocyclopropanecarboxylate oxidase. [file Image_3.TIF]

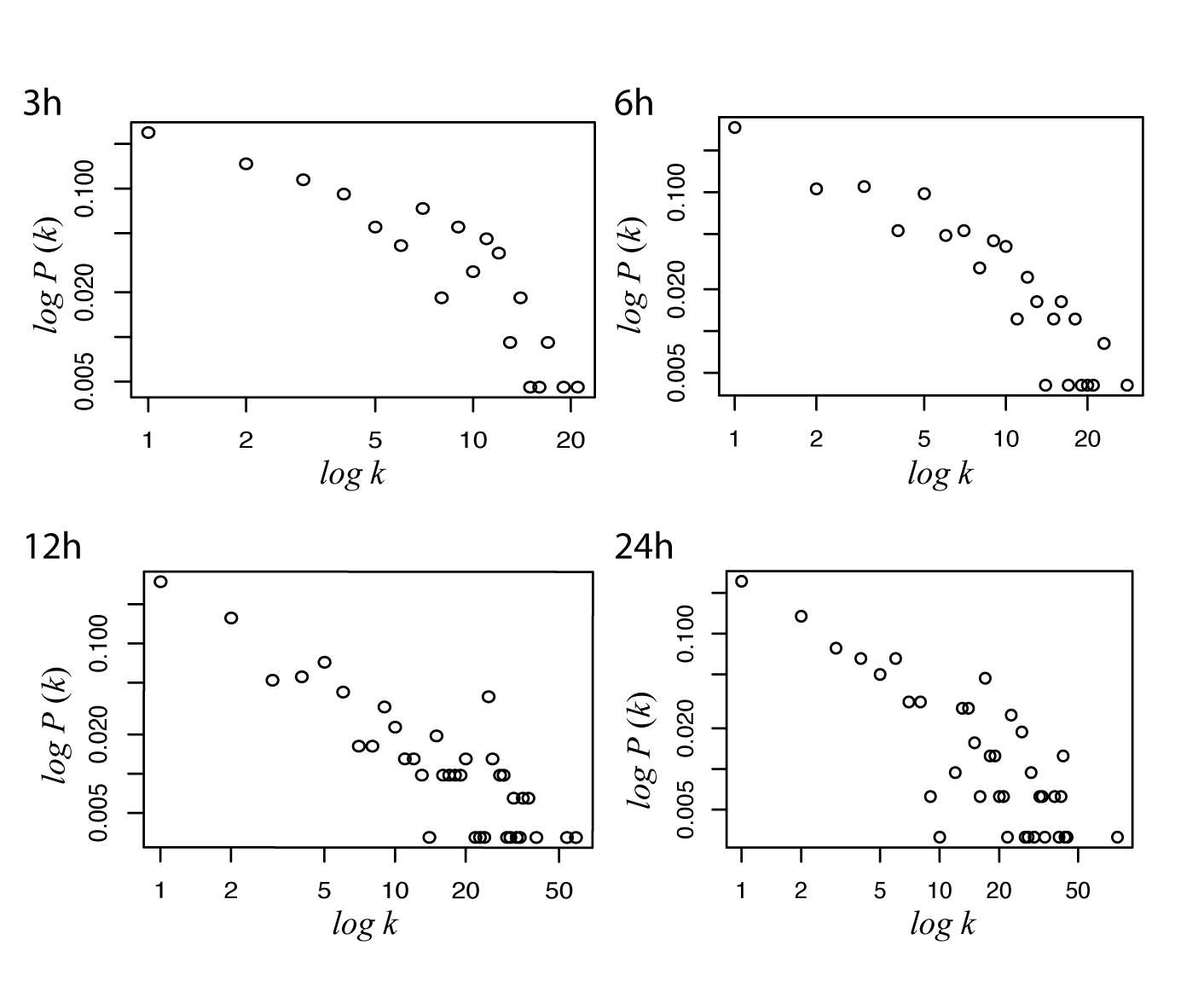

Supplement: Figure S4 — Log-log plots show a scatter plot of natural log of k against P(k) of the tripartite networks for each time point. [file Image_4.TIF]
